# Phase-Dependent Modulation of Signal Transmission in Cortical Networks through tACS-Induced Neural Oscillations

**DOI:** 10.3389/fnhum.2017.00471

**Published:** 2017-09-27

**Authors:** Kristoffer D. Fehér, Masahito Nakataki, Yosuke Morishima

**Affiliations:** ^1^Division of Systems Neuroscience of Psychopathology, Translational Research Centre, University Hospital of Psychiatry, University of Bern, Bern, Switzerland; ^2^PRESTO, Japan Science and Technology Agency, Saitama, Japan

**Keywords:** functional connectivity, effective connectivity, theta oscillations, transcranial magnetic stimulation, transcranial alternating current stimulation, electroencephalography

## Abstract

Oscillatory neural activity is considered a basis of signal transmission in brain networks. However, the causal role of neural oscillations in regulating cortico-cortical signal transmission has so far not been directly demonstrated. To date, due to methodological limitations, studies on the online modulatory mechanisms of transcranial alternating current stimulation (tACS)-induced neural oscillations are confined to the primary motor cortex. To address the causal role of oscillatory activity in modulating cortico-cortical signal transmission, we have established a new method using concurrent tACS, transcranial magnetic stimulation (TMS) and electroencephalography (EEG). Through tACS, we introduced 6-Hz (theta) oscillatory activity in the human dorsolateral prefrontal cortex (DLPFC). During tACS, we applied single-pulse TMS over the DLPFC at different phases of tACS and assessed propagation of TMS-induced neural activity with EEG. We show that tACS-induced theta oscillations modulate the propagation of TMS-induced activity in a phase-dependent manner and that phase-dependent modulation is not simply explained by the instantaneous amplitude of tACS. The results demonstrate a phase-dependent modulatory mechanism of tACS at a cortical network level, which is consistent with a causal role of neural oscillations in regulating the efficacy of signal transmission in the brain.

## Introduction

One of the most prominent features of brain activity is its oscillating pattern (Berger, 1929), which reflects network-wide, rhythmic changes in excitability (Thut et al., 2012). These oscillatory activities are increasingly considered fundamental for neuronal communication, and to enable flexible adjustments of signaling efficacy among relevant brain areas depending on the cognitive demands at the time (Engel et al., 2001; Varela et al., 2001; Buzsáki and Draguhn, 2004; Fries, 2005). The influence of the ongoing oscillatory phase on both neuronal functioning and sensory processing has long been recognized in animals (VanRullen et al., 2011). In humans, the variability of neural response and behavioral performance has been shown to depend on ongoing endogenous and experimentally induced neural oscillations (Dugué et al., 2011; Fellinger et al., 2011; VanRullen et al., 2011; Neuling et al., 2012; Riecke et al., 2015). In particular, the instantaneous phase of endogenous oscillations predicts transcranial magnetic stimulation (TMS) responsiveness (van Elswijk et al., 2010; Kundu et al., 2014), suggesting that the efficacy of neural transmission is related to the phase of neural oscillations.

Amidst vast observations on the role of oscillatory activity in modulating signaling efficacy, a causal association between the oscillatory phase and regional excitability has only recently been explored (Guerra et al., 2016; Nakazono et al., 2016; Raco et al., 2016), and its role in modulating transmission efficacy in neural networks has yet to be directly demonstrated. One approach to experimentally address the causal role of oscillatory activity in the brain is to use transcranial alternating current stimulation (tACS). tACS non-invasively applies a weak alternating (sinusoidal) current to the scalp (Paulus, 2011), which modulates the excitability of the cortex in a frequency-specific manner (Kanai et al., 2008; Zaehle et al., 2010; Polanía et al., 2012; Neuling et al., 2013; Helfrich et al., 2014a,b). Evidence for successful frequency-specific modulation of cortical activity using tACS has been reported post-stimulation, as well as during stimulation (Pogosyan et al., 2009; Zaehle et al., 2010; Neuling et al., 2013, 2015; Helfrich et al., 2014a,b; Witkowski et al., 2016; Violante et al., 2017), but little is known about the neurophysiological effects during stimulation. Direct demonstrations of a phase-dependent modulatory effect of tACS have been demonstrated in the primary motor cortex (Guerra et al., 2016; Nakazono et al., 2016; Raco et al., 2016), by means of concurrent motor-evoked potentials (MEPs) measures. While the scalp is known to effectively shunt the larger part of transcranially applied current (Opitz et al., 2016), these recent tACS-MEP studies have provided important evidence for the potency of tACS as a technique to non-invasively modulate neuronal excitability at a physiologically relevant magnitude.

However, relying on measures of MEPs, this approach cannot address regionally specific neurophysiological effects of tACS, i.e., beyond the primary motor cortex, or effects on transmission in cortico-cortical networks. To overcome these limitations, in the present study, we have established a new method using concurrent tACS, TMS and electroencephalography (EEG) to study the causal associations between oscillatory neural dynamics and signal transmission in neural networks. The rationale of the concurrent tACS-TMS-EEG method is as follows: while introducing extrinsic oscillations with tACS, we measure phase-dependent changes in signal transmission efficacy with the TMS-EEG technique. Single-pulse TMS to a cortical region induces neural activity that propagates through anatomically connected regions, and the direction and amount of current spread are modulated by the functional status of the neural network. Hence, measuring TMS-evoked potentials (TEPs) with EEG provides a measure of state-dependent changes in signaling efficacy (Ilmoniemi et al., 1997; Massimini et al., 2005; Driver et al., 2009; Morishima et al., 2009; Kundu et al., 2014). In the present study, we particularly focus on 6-Hz (theta-band) activity in the frontoparietal network, as cortical theta oscillations are associated with long-range cortico-cortical interactions (Polanía et al., 2012; Cohen, 2014; Violante et al., 2017). Such interactions are required during high-level cognitive processing and dynamically link prefrontal circuits to other task-related regions (Mizuhara and Yamaguchi, 2007; Sakai, 2008; Gregoriou et al., 2009; Liebe et al., 2012). Thus, our primary focus is phase-dependent transmission from the prefrontal cortex during theta-band tACS. Based on a previous study on the transient modulatory effects of short-duration transcranial direct current stimulation (tDCS; Nitsche and Paulus, 2000), we further hypothesize that cortical excitability will be enhanced at the 90° phase (crest) and suppressed at the 270° phase (trough) of the tACS-induced current.

## Materials and Methods

### Participants

Twenty-nine healthy human subjects participated in the study (14 females, mean age across subjects 24.3; range 19–38; all subjects right-handed). Of these, 18 subjects underwent tACS to the left prefrontal (dorsolateral prefrontal cortex, DLPFC) as well as to the left posterior parietal cortex (PPC). Owing to technical issues, the electronic current stimulators did not produce current output, and hence six subjects received tACS only to the DLPFC, and five subjects received tACS only to the PPC. Due to insufficient data quality, seven subjects were excluded in total: three subjects undergoing frontal tACS, three subjects undergoing parietal tACS and one subject undergoing both parietal and frontal tACS. The reasons for rejecting their data were the presence of a large amount of residual tACS-induced and/or TMS-induced artifacts. As excessive blinking artifacts, muscle artifacts, or a combination of these made it difficult to efficiently remove tACS-induced and/or TMS-induced artifacts for these subjects, too few trials were left to reliably analyze the data of these subjects. Finally, data from 20 subjects receiving frontal tACS and 19 subjects receiving parietal tACS were subjected to further analysis. All participants provided written informed consent and were screened for contraindications to TMS (Rossi et al., 2011), as well as for a previous history of mental illness or neurological disorders prior to enrolment. The study was carried out in accordance with the Declaration of Helsinki, and the study protocol was approved by the ethics committee of Canton Bern (KEK-BE 007/14). Written consent was obtained from all participants in accordance with the Declaration of Helsinki.

### TMS Procedure

TMS with a biphasic current waveform was administered with a figure-eight coil with a diameter of 75 mm (MCF-B65 Butterfly Coil, Magventure A/S, Denmark) connected to a MagPro R30 magnetic stimulator (Magventure A/S, Denmark). The intensity of single-pulse TMS was set to 40% of the maximal output intensity of the stimulator. This corresponded to an average of 76.4% of the active motor threshold, as measured by finger twitch. The active motor threshold was measured before the main measurements, while the subject was wearing the EEG cap to take into account the additional space between the coil and the scalp caused by the EEG cap.

### Concurrent tACS-TMS-EEG Recording

**Figure 1:**
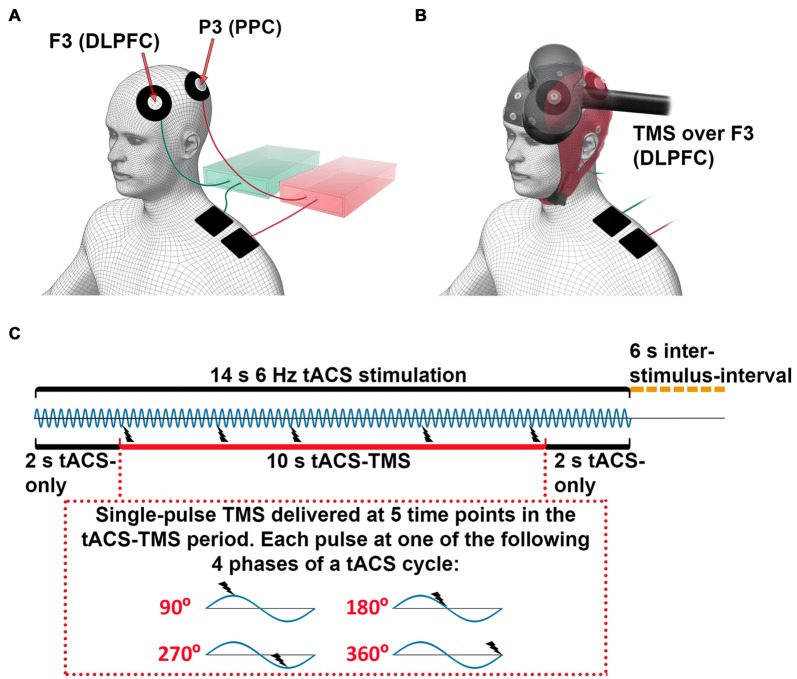
Experimental setup for concurrent transcranial alternating current stimulation (tACS)-transcranial magnetic stimulation (TMS)-electroencephalography (EEG) recording. **(A)** tACS electrode configuration: each electronic current stimulator was connected to one scalp electrode, centered at channels F3 (dorsolateral prefrontal cortex, DLPFC) or P3 (posterior parietal cortex, PPC) and one return electrode was placed on the ipsilateral shoulder. For illustration purposes, the stimulators and the cables to their respective coupled scalp and return electrodes are marked with the same color (green and red). **(B)** tACS-EEG-TMS montage: TMS was applied over the F3 electrode (DLPFC). **(C)** Timeline of stimulation. The 6-Hz tACS was delivered in blocks of 14 s, followed by 6-s inter-stimulus-intervals. During one tACS block, a single-pulse TMS was applied at 5 time points between 2 s and 12 s after the onset of tACS. The inter-TMS-interval was jittered between 2 s and 3.5 s. Each single-pulse TMS was applied at one of four different phases of a tACS-cycle (90°, 180°, 270°, 360°)—that is, three TMS-delivery phases occurred only once per tACS-block, while one TMS-delivery phase occurred twice. The order of TMS-delivery phases was randomized.

During the concurrent tACS-TMS-EEG recordings, the TMS coil was placed tangential to the scalp, centered over electrode F3 (which corresponds to the middle frontal gyrus; Koessler et al., 2009) with the handle pointing to the left and 45° away from the midline (Figure 1B). Electrophysiological data were recorded with a 24-bit EEG amplifier (eego sports, ANT Neuro, Netherlands) using 32 Ag/AgCl electrodes (waveguard, ANT Neuro, Netherlands). The recording reference was located at CPz, and the ground electrode was located at AFz. EEG electrodes were placed according to the international 10–20 system for EEG electrode positioning (Jasper, 1958). EEG data were sampled at 2048 Hz. The impedance of each EEG electrode was kept below 5 kΩ.

Subjects were seated in a dimly lit room and were instructed to relax, fixate on a point in front of them, and keep still during the recording and stimulation. Subjects were also instructed to wear earplugs during measurements. Head movements were restricted by a chin-rest, and the position of the TMS coil over the scalp was fixed with a tripod to ensure constant coil placement throughout the experiment.

We recorded eight sessions of concurrent tACS-TMS-EEG. For the subjects who received tACS only at the DLPFC or PPC, the experiment consisted of only four tACS-TMS-EEG sessions. Each of the tACS-TMS-EEG sessions lasted for approximately 6 min, between which the subjects could take a short break. tACS was delivered through two electronic current stimulators (DC-Stimulator plus, NeuroConn GmbH, Germany). Each stimulator was connected to one doughnut-shaped stimulating electrode placed on the scalp (outer diameter, 60 mm; inner diameter, 25 mm; surface area, 23.4 cm^2^) and one return electrode (50 × 50 mm; surface area, 25 cm^2^) placed on the ipsilateral shoulder of the participant. The scalp electrodes were centered at channels F3 (DLPFC) and P3 (PPC; Figure 1A). The tACS electrode montages were selected by means of electric field modeling (Supplementary Figure S1). The tACS electrodes were made of conductive rubber (NeuroConn GmbH, Germany) and attached to the scalp with EEG gel, as we have described in a previous protocol article (Fehér and Morishima, 2016). The impedances of the tACS electrodes were kept below 10 kΩ. A sinusoidal current was applied with the tACS, with a peak-to-peak intensity of 0.9 mA at a frequency of 6 Hz.

In each of the eight (or four) recording sessions, tACS was delivered in 16 blocks. Each tACS block lasted for 14 s followed by 6-s inter-stimulus-intervals (Figure 1C). For subjects receiving tACS to both the DLPFC and the PPC, tACS was delivered to the DLPFC for half of the stimulation blocks and to the PPC for the other half of the stimulation blocks. The order of these two types of tACS blocks was randomized. During each tACS-block, a single TMS-pulse (at 40% of maximal output intensity) was applied at 5 time points, with the first and last pulse of each tACS-block delivered 2 s after the onset and before the offset of tACS, respectively. The inter-TMS-interval within a tACS-block was jittered between 2–4 s (i.e., 12–24 tACS-cycles). Each TMS-pulse was applied at one of four different phases of a tACS-cycle (90°, 180°, 270°, 360°; Figure 1C). Within a tACS-block, each TMS-delivery phase occurred once, except one delivery phase, which occurred twice. The order of the TMS-delivery phases within one tACS-block and within one recording session was randomized. During each recording session, a total of 80 TMS-pulses were delivered, with each TMS-delivery phase occurring an equal amount of times. In total for eight sessions, 640 pulses were delivered, with 80 TMS trials per condition (frontal tACS 90°, 180°, 270°, 360°, parietal tACS 90°, 180°, 270° and 360°). Subjects who received tACS only to the DLPFC or the PPC went through four sessions with 320 TMS trials (80 TMS trials per condition). An analog output board (model NI PCI-6723, National Instruments, Austin, TX, USA) was used to control the tACS stimulators and the TMS device and to send triggers to the EEG system. The output board was controlled through the data acquisition toolbox for MATLAB (MathWorks Inc., Natick, MA, USA). The tACS stimulator, the TMS system, and the triggers for the EEG system were controlled via separate analog channels, sending a sinusoidal waveform to the tACS stimulator to control current output, and 5V signals to the TMS system to trigger a TMS pulse and to record timings in the EEG system. In this way, we controlled the timing of the TMS pulse in relation to the specific tACS phase and recorded the onset of tACS and TMS.

### tACS Artifact Removal

**Figure 2:**
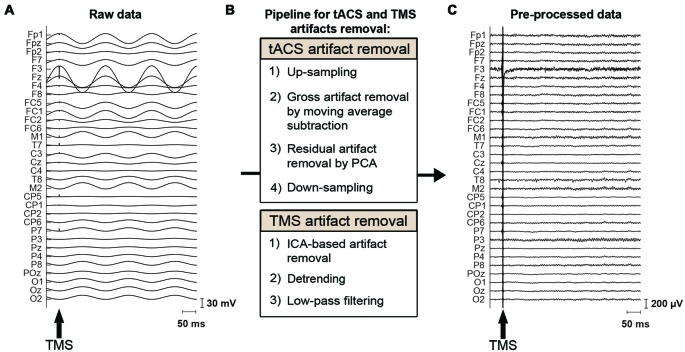
tACS and TMS artifact removal procedure. **(A)** Raw EEG data, contaminated by the tACS- and TMS-induced artifacts. The magnitude of the tACS artifact can exceed 100 mV in the vicinity of a stimulation electrode when 0.9 mA tACS is applied to the left DLPFC (F3). The arrow indicates the onset of TMS. **(B)** Pipeline for removal of the tACS- and TMS-induced artifacts. To remove the tACS artifact, EEG data are first up-sampled to adjust trigger timing. The major part of the tACS artifact is removed by moving-average subtraction of tACS-cycles. Residual small and periodic artifacts are removed by principal component analysis (PCA). Finally, the data are again down-sampled before further processing. To remove the TMS artifacts, TMS artifact related components are removed through independent component analysis (ICA). The data is then detrended and finally low-pass filtered before further analysis. **(C)** The same section of EEG data as shown in **(A)** after removal of the tACS- and TMS-induced artifacts.

EEG data were preprocessed using MATLAB and the EEGLAB toolbox (Delorme and Makeig, 2004)^1^. The characteristics of the tACS-related artifact are similar to the MRI gradient-related artifact on EEG data: a periodic artifact with large amplitude. We therefore adapted the MRI artifact removal pipelines from EEG data (Negishi et al., 2004; Niazy et al., 2005) to our tACS-induced artifact removal process (Figure 2). This approach has been used in recent studies employing concurrent tACS-EEG (Helfrich et al., 2014b). The data were first up-sampled to 9600 Hz to adjust trigger timing. The majority of the tACS artifact was then removed per channel using moving average subtraction. The moving average window included 16 tACS-cycles, from which a mean cycle was calculated. tACS cycles including a TMS pulse were not included in the moving average but were interpolated by the adjacent tACS cycles. That is, here we made an average of eight cycles prior to and eight cycles following the tACS cycle that included a TMS pulse. The moving average was calculated starting at 45°, 135°, 225° or 315° of the tACS cycle, and an average of this was subtracted from the data. Following the moving average subtraction, the TMS-trigger timing was adjusted to the up-sampled data. Residual small and periodic artifacts were then removed by principal component analysis (PCA), applied per channel (Supplementary Figure S2). This differs from the aforementioned approach used by Helfrich et al. (2014b) where a spatial PCA was instead performed over all channels. As for the moving average subtraction, removing components of the tACS cycles that included a TMS-pulse delivery were interpolated by the adjacent tACS cycles. Here we made an average of 1 cycle prior to and following the tACS cycle that included a TMS pulse. Finally, the data were again down-sampled to 2000 Hz.

### TMS Artifact Removal

**Figure 3:**
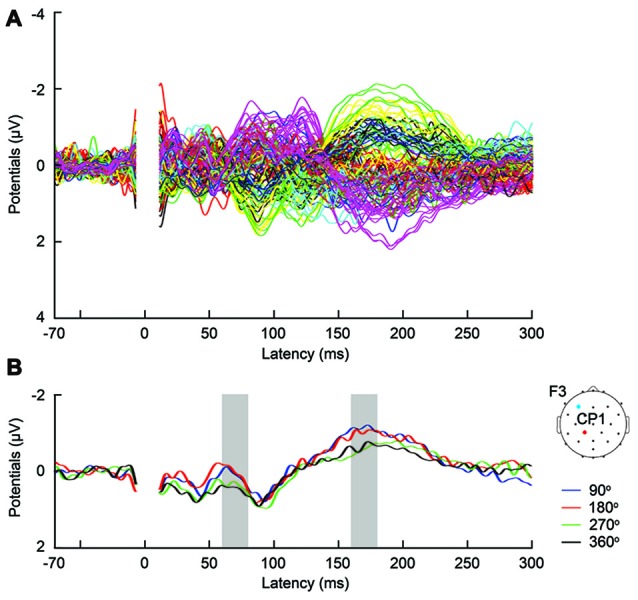
**(A)** Butterfly plot of grand-average TMS-evoked potentials (TEPs) across participants during frontal tACS, for all channels and TMS conditions. **(B)** Example channel CP1 after artifact removal, with grand-average TEPs across participants, per TMS-delivery phase during frontal tACS. Channel CP1 is marked in red on the head model. The tACS electrode was centered on electrode F3 and is marked in blue on the head model. The shaded areas show time windows with significant effect of phase (*p* < 0.05) on TEPs based on ANOVA (60–80 and 160–180 ms, respectively after TMS).

For the removal of TMS induced artifacts, we adapted the pipeline suggested by Rogasch et al. (2017; Figure 2). The tACS-artifact cleaned data was segmented into epochs (−200, 500 ms) with respect to the TMS onset. Channels F3 as well as the mastoids were excluded at this stage from the data. At channel F3, long average TMS artifact decay prevented complete removal of tACS-related artifacts. Channel F3 was furthermore highly contaminated with random noise unrelated to the application of TMS. The mastoids were excluded as tACS-related artifacts in the EEG data were not effectively removed due to muscle artifacts across subjects. At this stage, we visually inspected the tACS-artifact cleaned data and found seven subjects for which the artifact removal was unsuccessful, resulting in an insufficient number of good trials left to reliably analyze their data. We accordingly excluded those subjects from further data processing and analyses. Among the remaining 20 subjects receiving frontal tACS and 19 subjects receiving parietal tACS that were included in our analyses, trials contaminated with large amplitude artifacts were removed by visual inspection. On average across these subjects 91.43% of the trials (i.e., 73.14 trials per TMS-delivery condition) entered further processing and analyses (Supplementary Table S1). TMS-related artifacts were first removed from the data using independent component analysis (ICA; FastICA; Hyvärinen and Oja, 2000). To calculate the ICA weights, the data was first merged across TMS conditions, the TMS-pulse period (−7, 10 ms) was removed, and the data was downsampled to 500 Hz. Components including the decaying TMS-artifact were selected manually, and the calculated weights were then applied back to each TMS condition of the data with the original sampling rate of 2000 Hz. On average across subjects, 2.1 components (SD = 0.8) were removed from the frontal tACS data, and 2.2 components (SD = 1.1) from the parietal tACS data. Following ICA, the period 10–500 ms with respect to the TMS onset was linearly detrended. The data was subsequently low-pass filtered (zero-phase, fourth order Butterworth low-pass) at 100 Hz. Finally, the artifact-cleaned data was then re-referenced to the average of all electrodes (excluding F3 and the mastoids). Baseline correction was performed with the −70 to −10 ms period with respect to the TMS onset. We also excluded the first 20 ms after TMS from the analysis to avoid TMS artifacts (Figure 3).

### EEG Data Analysis

Statistical analyses were performed on TEPs in MATLAB and in R (R Development Core Team, 2008)^2^. Phase-dependent differences in TEPs were analyzed separately for each of the frontal or parietal tACS data set. One-way repeated measures ANOVAs were calculated channel-wise for mean TEPs across time windows of 20 ms after TMS. Due to excessive artifacts, channel F3 and mastoids were not included in any statistical analyses. We used the frontal tACS data set to assess the basic assumptions of a linear relationship between applied current and excitability. To this end, we performed planned pair-wise comparisons of TEPs between the 90° and 270° phases as well as between the 180° and 360° phases of tACS, calculated channel-wise for mean TEPs across time windows of 20 ms after TMS. A significance threshold of 0.05 was used for all statistical analyses.

In order to assess whole brain topographic changes, phase-dependent differences in TEPs were also analyzed, separately for the frontal and parietal tACS data sets, through one-way repeated measures MANOVAs (car package; Fox and Weisberg, 2011), for mean TEPs across time windows of 20 ms after TMS. Channel (29 levels) and phase (4 levels) were treated as within-subject factors. Another motivation for performing MANOVAs was to confirm the findings from our channel-wise ANOVAs while avoiding the false positive inflation that comes with channel-wise analyses. For the frontal tACS data set, we also compared TEPs between the 180° and 360° phases of tACS through one-way repeated measures MANOVAs, for mean TEPs across time windows of 20 ms after TMS. Channel (29 levels) and phase (2 levels) were treated as within-subject factors. A significance threshold of 0.05 was used for all statistical analyses.

In order to obtain the overall modulation of TEPs across channels, we also calculated the global mean field power (GMFP; Lehmann and Skrandies, 1980) from the multichannel average signals as follows:

$$\text{GMFP}(t)=\sqrt{\frac{\sum_{i}^{k}{\left(V_{i}(t)-V_{\text{mean}}(t)\right)}^{2}}{k}}$$

GMFP was calculated from the baseline-corrected TEPs data for each subject and each tACS phase. Phase-dependent differences in the GMFP were analyzed separately for each of the frontal or parietal tACS data set through one-way repeated measures ANOVAs, calculated for mean GMFP across time windows of 10 ms after TMS. To assess the basic assumptions of a linear relationship between applied current and the global modulation of excitability, we also compared GMFP between the 180° and 360° phases of tACS, separately for the frontal and parietal tACS data sets. The planned two-tailed paired *t*-tests were performed for mean GMFP across time windows of 20 ms after TMS.

## Results

### Phase-Dependence of TEPs during Frontal tACS

**Figure 4:**
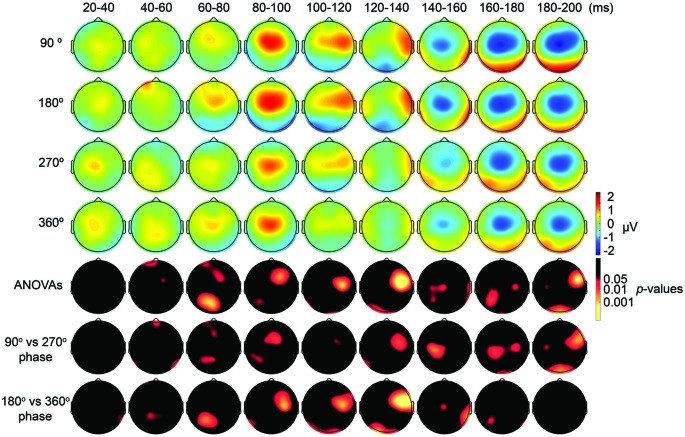
Scalp maps of TEPs during frontal tACS showing mean across participants, and statistical analyses. **Top four rows**: scalp topographies of TEPs between 20 ms and 200 ms after TMS during 6-Hz tACS applied to the DLPFC. TMS was applied at 90°, 180°, 270° or 360° of the tACS phase. Channel F3 and mastoids were excluded from the plot, and the potentials were interpolated from surrounding channels. **Fifth row**: scalp topographies across time of *p*-values based on channel-wise one-way repeated measures ANOVA for the effect of the delivery-phase of the TMS. **Sixth row**: scalp topographies across time of *p*-values based on a channel-wise comparison between 90° and 270° tACS phase. Channel F3 and mastoids were excluded from statistical analyses. The *p*-value of each electrode is shown in color only when it reached significance (*p* < 0.05). **Seventh row**: scalp topographies across time of *p*-values based on a channel-wise comparison between 180° and 360° tACS phase. Channel F3 and mastoids were excluded from statistical analyses. The *p*-value of each electrode is shown in color only when it reached significance (*p* < 0.05).

First, we expected that 6-Hz tACS to the DLPFC would cause phase-dependent changes in excitability in the DLPFC and that TMS at the 90° and 270° phases of tACS would induce larger and smaller TEPs, respectively. After removal of the tACS- and TMS-induced artifacts (Figure 2, Supplementary Figure S2), we calculated condition-specific TEPs and compared TEPs among TMS delivery phases. Interestingly, the ANOVA yielded significant differences in TEPs between TMS-delivery phases in frontal electrodes, beginning from the time window of 40–60 ms after TMS (*p* < 0.05). In later time windows, we also found that significant phase-dependent differences in TEPs emerged in posterior and anterior regions and then propagated to contralateral regions (Figure 4, *fifth* row). The MANOVA reiterated these results, showing a significant interaction effect of phase and channel, starting from the time window of 40–60 ms after TMS (*F*_(84,1596)_ = 1.590, *p* = 0.0007), while a significant main effect of phase alone was only observed from the time window of 220–240 ms after TMS (*F*_(3,57)_ = 2.915, *p* = 0.042; Supplementary Table S2). As a planned *post hoc* comparison, we then looked at the differences in TEPs when TMS was applied at the 90° or 270° phase of tACS. We observed a significant difference starting from the time window of 40–60 ms after TMS at channels Fpz (*p* = 0.013), P7 (*p* = 0.033) and P8 (*p* = 0.008; Figure 4, *sixth* row). These results suggest that 6-Hz tACS can modulate cortical excitability in a phase-dependent manner.

### Phase-Dependence of TEPs during Parietal tACS

**Figure 5:**
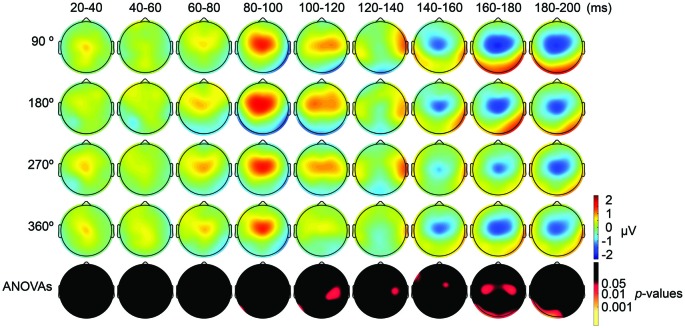
Scalp maps of TEPs during parietal tACS showing mean across participants, and statistical analyses.** Top four rows**: scalp topographies across time of TEP amplitude at 20–200 ms after TMS during 6-Hz tACS applied to the PPC. Mastoids and channel F3 were excluded from the plot, and the potentials were interpolated from surrounding channels. **Fifth row**: scalp topographies across time of *p*-values based on channel-wise one-way repeated measures ANOVA for the effect of the delivery-phase of the TMS. The mastoids and channel F3 were excluded from any statistical analysis. The *p*-value of each electrode is shown in color only when it reached significance (*p* < 0.05).

We also applied tACS to the PPC and measured TEPs when TMS was applied to the DLPFC. Our motivation for applying tACS to the PPC was to ensure that the artifact removal pipeline did not create false-positive differences among TMS-delivery conditions. Namely, we consider that TMS-phase dependence should arise at least 20 ms later during parietal tACS as compared to during frontal tACS, as the TMS induced signal first would need to propagate from the DLPFC to the PPC. After TMS-induced activity reaches the PPC, TMS-induced activity interacts with the PPC activity modulated by tACS. Consistent with our expectations, the ANOVA yielded no significant differences in TEPs between TMS-delivery phases (*p* > 0.05; Figure 5, *fifth* row) in the earlier time windows (up to 80 ms after TMS) and, as expected, significant differences in TEPs between the four TMS-delivery phases were observed in later time windows (> 80 ms after TMS). The ANOVA revealed significant phase dependence starting from the 80–100 ms time window after TMS (Figure 5, *fifth* row) at channel P7 (*F*_(3,18)_ = 2.838, *p* = 0.047). The MANOVA showed a significant interaction effect of phase and channel, starting from the time window of 100–120 ms after TMS (*F*_(84,1512)_ = 1.543, *p* = 0.0015), while a significant main effect of phase alone was not observed (*p* > 0.05). These results confirm that the phase-dependent results in the current study are not a product of tACS-induced artifacts but are instead bona fide neurophysiological changes. Namely, the results suggest that when applying tACS to the PPC, the phase-dependent modulation of TEPs occurred only after the TMS-induced signal had propagated from the DLPFC to the PPC. In contrast, when tACS was applied to the DLPFC, the phase-dependent modulation of TEPs was observed in earlier components after TMS.

### Modulatory Mechanism of tACS

The results of our analyses of TEPs during frontal tACS suggested that 6-Hz tACS can modulate cortical excitability in a phase-dependent manner. Based on the transient modulatory effects of short-duration tDCS on cortical excitability (Nitsche and Paulus, 2000), we can hypothesize that the applied current and the resulting changes in excitability levels exhibit a linear relationship, as shown in a recent tACS-MEP study (Nakazono et al., 2016). While the 90° (crest) and 270° (trough) phases of the 6-Hz tACS would yield the highest and lowest levels of excitability, respectively, no differences would then be expected between the 360° and 180° phases. The observed differences in TEPs when TMS was applied at the 90° or 270° phase of tACS are consistent with this prediction. We assessed the differences in TEPs when TMS was applied at the 180° or 360° phase of tACS. Contrary to what we had expected, channel-wise comparisons yielded significant differences between the conditions, in the time window 20–40 ms after TMS at channel P8 (*p* = 0.046), and from the time window 40–60 ms after TMS at channels Fp1 (*p* = 0.047) and CP1 (*p* = 0.039; Figure 4, *seventh* row), with overall magnitudes of differences similar to the 90° and 270° phases, respectively (Figure 4). Multivariate comparisons between the 360° and 180° phases also showed a significant interaction effect of phase and channel, starting from the time window of 60–80 ms after TMS (*F*_(28,532)_ = 1.903, *p* = 0.004), while a significant main effect of phase alone was only observed from the time window of 100–120 ms after TMS (*F*_(28,532)_ = 5.320, *p* = 0.033). These results suggest that the assumption of a linear relationship between the applied current amplitude and the resulting excitability level does not hold for 6 Hz tACS.

**Figure 6:**
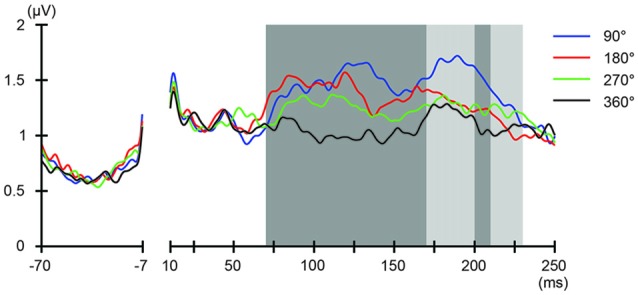
Phase-dependent modulation of excitability during frontal tACS as measured by global mean field power (GMFP). The onset of TMS corresponds to 0 ms of the plot. The shaded areas mark time windows with significant effect (*p* < 0.05) of phase on GMFPs during frontal tACS (70–230 ms after TMS), based on one-way repeated measures ANOVA for the effect of the delivery-phase of the TMS. ANOVAs were calculated for mean GMFPs across time windows of 20 ms after TMS. The mastoids and channel F3 were excluded from any statistical analysis. The darker shaded areas additionally mark time windows with significant differences (*p* < 0.05) between 180° and 360° delivery-phase of the TMS in GMFP, during frontal tACS (70–170 and 200–210 ms, respectively after TMS), based on planned paired *t*-test between 180° and 360° delivery-phase of the TMS. *T*-tests were calculated for mean GMFPs across time windows of 20 ms after TMS. The mastoids and channel F3 were excluded from any statistical analysis.

**Figure 7:**
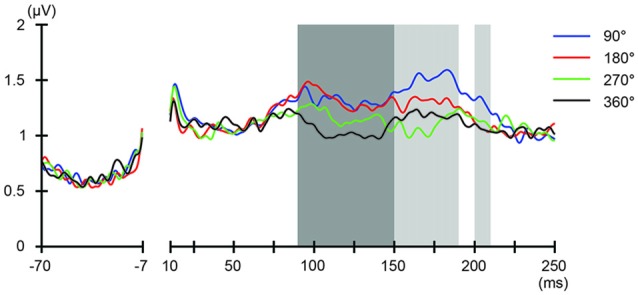
Phase-dependent modulation of excitability during parietal tACS as measured by GMFP. The onset of TMS corresponds to 0 ms of the plot. The shaded areas mark time windows with significant effect (*p* < 0.05) of phase on GMFPs during parietal tACS (90–190 and 200–210 ms, respectively after TMS), based on one-way repeated measures ANOVA for the effect of the delivery-phase of the TMS. ANOVAs were calculated for mean GMFPs across time windows of 20 ms after TMS. The mastoids and channel F3 were excluded from any statistical analysis. The darker shaded areas additionally mark time windows with significant differences (*p* < 0.05) between 180° and 360° delivery-phase of the TMS in GMFP, during parietal tACS (90–150 ms after TMS). Based on planned paired *t*-test between 180° and 360° delivery-phase of the TMS. *T*-tests were calculated for mean GMFPs across time windows of 20 ms after TMS. The mastoids and channel F3 were excluded from any statistical analysis.

We also addressed the relationship between the applied current and the global modulation of excitability, by calculating the GMFP for TEPs obtained during frontal and parietal tACS. We calculated condition-specific GMFPs and compared TEPs among TMS delivery phases (Figures 6, 7). The one-way repeated-measures ANOVA yielded significant differences in GMFPs between TMS-delivery phases during frontal tACS, beginning from the time window of 70–80 ms after TMS (*F*_(3,19)_ = 3.938, *p* = 0.0127; Figure 6), and during parietal tACS, beginning from the time window of 90–100 ms after TMS (*F*_(3,19)_ = 4.092, *p* = 0.0109; Figure 7). We then assessed the differences in TEPs when TMS was applied at the 180° or 360° phase of tACS. The planned pair-wise comparison yielded significant differences between the conditions during frontal tACS, in the time window 70–80 ms after TMS (*p* = 0.0024; Figure 6). This is consistent with the channel-wise comparisons of the 180° or 360° phase and contrary to the assumptions of a linear relationship between applied current changes in excitability levels. These results again suggest that the assumption of a linear relationship between the applied current amplitude and the resulting excitability level does not hold. It also reiterates the results obtained from the *post hoc* comparison of TEPs when TMS was applied at the 180° or 360° phase of tACS.

## Discussion

We examined whether tACS can modulate cortico-cortical signaling efficacy in a phase-dependent manner. Despite recent enthusiastic use of tACS to introduce oscillatory activity, few studies have addressed the modulatory mechanism of tACS, or directly assessed the potency of tACS as a tool for modulating oscillatory activity. In the current study, we established the concurrent tACS-TMS-EEG method to address the effect of tACS-induced neural oscillation on cortical signal transmission. First, we have shown that tACS can modulate cortical transmission in a phase-dependent manner. Second, the modulatory mechanism of tACS is not simply explained by the instantaneous amplitude of applied current.

Our experiments consisted of frontal and parietal tACS. During frontal tACS, we introduced 6-Hz activity in the area over which TMS was applied. Whereas enhancement of theta activity that outlasts the offset of theta frequency tACS is controversial (Veniero et al., 2015), we show that our short-duration theta tACS—ranging from 2 s to 12 s before a TMS pulse (Figure 1C)—is enough to modulate cortico-cortical signal transmission in a phase-dependent manner during stimulation. In addition, it has previously been demonstrated that 4 s of tDCS can enhance or suppress cortical excitability respectively during stimulation, depending on the current polarity (Nitsche and Paulus, 2000). Thus, instantaneous modulation can be achieved in tACS. However, sustained enhancement of oscillatory activity at the applied frequency is less likely to be achieved, if the applied tACS frequency is deviating from the endogenous frequency.

The magnitude of TEP differences among TMS timings in the current experiments is 0.5–1 μV (Figure 3B, Supplementary Figure S3), which is comparable to our previous TMS-EEG studies on task-dependent changes in signal transmission (Morishima et al., 2009; Akaishi et al., 2010). The results suggest that the potency of the modulatory effect of tACS would be equivalent to endogenously modulated network states. These findings provide strong experimental support for the role of endogenous oscillatory activity in modulating cortical excitability and signaling efficacy.

The results obtained during parietal tACS substantiate the main results obtained during frontal tACS. Consistent with our expectations, during parietal tACS, we observed phase-dependent differences between TEPs after TMS to the DLPFC to only arise after some considerable delay, reflecting that we applied tACS not to the region under the TMS but to the parietal cortex. Therefore, phase-dependent differences would first arise in later time windows, after propagation of the TMS-induced signals from the DLPFC to the PPC. In agreement with these expectations, the phase-dependent variation of TEPs between the four TMS-delivery phases during parietal tACS were observed beyond 80 ms after TMS. The magnitudes of phase-dependent variation were furthermore smaller compared to the magnitudes of variation observed during frontal tACS. Two possible mechanisms can explain the smaller phase-dependent variations of TEPs. First, during frontal tACS, the activity of most of the neurons stimulated by TMS was also modulated by tACS. However, during parietal tACS, only the TMS-stimulated neurons that project from the DLPFC to the PPC may be interacting with neurons modulated by tACS in the PPC. Second, as we only controlled the phase-state in the PPC, the TMS-induced signal was transmitted from the DLPFC at a mix of more or less ideal phase-states. Averaging across such TEPs could therefore be expected to result in an attenuated phase-dependent variation of TEPs. In conclusion, the results after parietal tACS indicate that the artifact removal pipeline did not create false-positive differences among TMS-delivery conditions, verifying the validity of the main results obtained during frontal tACS.

One limitation of the current study is that we did not include a separate sham tACS condition, i.e., TMS-only condition, to directly compare TEPs with and without concurrent tACS. Inclusion of such a condition might for instance have provided us with a baseline of TMS-evoked EEG potentials, from which we could have inferred an absolute direction of phase-dependent excitability changes, i.e., whether excitability was up- or down-regulated.

Both during the parietal and frontal tACS conditions, the durations before we could observe significant phase-dependent changes in excitability were longer than we had expected. During the frontal tACS condition, we applied TMS over the region modulated by the tACS. Previous studies on human cortico-cortical interactions suggest that we would expect to see the first phase-dependent differences in TEPs in the time window of 20–40 ms after TMS (Pascual-Leone and Walsh, 2001; Silvanto et al., 2006; Matsumoto et al., 2007; Morishima et al., 2009; Akaishi et al., 2010). However, in the frontal tACS condition we observed significant phase-dependent differences in TEPs at 40–60 ms after TMS. In the parietal condition, we would similarly have expected to see phase-dependent differences in TEPs with an additional delay of 20–40 ms. We observed significant phase-dependent differences in TEPs at 80–100 ms after TMS. While the relative time difference in the onset of phase-dependent effects between the frontal and parietal conditions was still consistent with our prediction (Figure 4 *fifth* row, Figure 5 *fifth* row, Figures 6, 7), the onsets of phase-dependent effects were delayed in both conditions. The apparent general delay might have several explanations. First, our experimental setup has a lower signal-to-noise ratio compared with TMS-EEG experiments without tACS, meaning that it might not have been sensitive enough to detect initial, weak phase-dependent changes. Second, our short-duration theta tACS might have been modulating excitability with variable efficacy. The first TMS pulse was applied after 2 s of tACS, while the last TMS pulse was applied after 12 s of tACS. Therefore, this might also have decreased the signal-to-noise ratio. Unfortunately, it would not be possible with our data to assess the relationship between tACS duration before the TMS-pulse and the magnitude of phase-dependent modulation in cortical excitability, as there were at most 16 trials for each TMS timing when comparing between the first and the last TMS pulse of the 14 s tACS blocks. This factor should however be systematically addressed in future studies.

One limitation of the current experimental settings is the use of a fixed stimulation sites across our subjects, placing the tACS electrodes and positioning the TMS coil according to the international 10–20 system for EEG electrode positioning. Use of neuronavigation would have allowed for more precise targeting of the stimulation site and would have informed us about the individual variation of the location of the TMS coil position in relation to the DLPFC. This in turn would have provided further information regarding individual variation in the TEPs response. However, it would have constituted a problem to implement individual tACS electrode montages and TMS coil positioning in the current experiments. As electrical bridging between tACS electrodes and EEG electrodes would clip the data recorded at the corresponding EEG channels, as reported previously (Fehér and Morishima, 2016), altering the location of the tACS electrodes would have meant inclusion and exclusion of different EEG electrodes across individuals. In addition, in order to assess tACS-phase dependent changes in local cortical excitability in the DLPFC, it was crucial that the tACS and TMS was applied over the same location, and hence the TMS coil placement was defined by the frontal tACS electrode position centered at channel F3.

In the current study, TMS intensity was fixed at 40% of the maximal output intensity of the stimulator, in contrast to the more conventional approach of adjusting the intensity of the TMS to the individual motor threshold. The reason for choosing to use a fixed TMS intensity was that we observed that high intensity TMS introduced severe artifacts in the EEG data, which could affect the performance of our artifact removal procedure. Consequently, we decided to keep the intensity fixed across subjects in order to avoid inhomogeneous performance of our artifact removal procedure. However, we cannot exclude the possibility that the individual motor threshold might have influenced the observed phase-dependent effect. Therefore, in order to test for this possibility, we divided our participants according to their motor threshold into two groups, either high or low MT group. We assessed the possibility that the channels and time-windows which had exhibited significantly phase-dependent TEPs (*p* < 0.05) during frontal tACS (Figure 5, *fifth* row), were influenced by the individual motor threshold. For these channels and time-windows, we calculated channel-wise two-way ANOVAs for mean TEPs across time windows of 20 ms after TMS for the frontal data set, with motor threshold (2 levels) treated as a between-subject factor, and phase (4 levels) as a within-subject factor. We identified only one channel and time-window during frontal tACS which showed a significant interaction effect between phase and motor-threshold, at channel Oz, 160–180 ms after TMS (*p* = 0.048). It is therefore unlikely that individual differences in motor threshold would underlie the phase-dependent results observed in this study. It should be noted however, that these analyses only included 10 subjects in each motor threshold group, and therefore might not have been powerful enough to detect a possible weak influence of individual motor threshold on the observed phase-dependent results.

Some of the factors that have been highlighted by previous studies to dictate the efficacy of driving ongoing intrinsic oscillatory activity include the momentary brain state, site-specific heterogeneities in the power-spectrum, and as well as individual heterogeneities in intrinsic oscillatory activity. First, tACS has been shown to have different efficacy depending on e.g., the task performed by the subject while stimulated, (Feurra et al., 2013; Kar and Krekelberg, 2014), or whether the subjects had their eyes open or closed (Neuling et al., 2013; Alagapan et al., 2016), suggesting a state-dependent efficacy of entrainment in response to tACS. Second, each cortical area has a unique anatomical architecture and functional properties. In particular, each cortical area has its own unique pattern of power spectrum (Supplementary Figure S4) as well as natural frequency induced by high intensity TMS (Rosanova et al., 2009), suggesting that the responsiveness to tACS of a particular frequency will differ among brain areas. For instance, we calculated the power-spectrum of resting-state EEG data for each subject. The channel-wise power-spectrum showed a higher alpha power occipitally (channel Oz) while theta power was higher at channel F3 (Supplementary Figure S4) over which our frontal tACS scalp electrode was centered. Such heterogeneity of intrinsic oscillatory activities might affect the responsiveness to tACS. Future studies are awaited to elucidate inter-regional specificities. Finally, individual differences in the intrinsic frequency of endogenous oscillatory activity has been suggested to render a particular frequency of stimulation more or less efficient in driving the intrinsic frequency. All these three parameters define the responsiveness to tACS, however not in an all-or-none manner. Mathematical models have shown that the efficacy of oscillatory stimulation to entrain ongoing oscillatory activity stand in an Arnold tongue relationship to on the one hand the mismatch between the applied and the endogenous frequency, and on the other hand the amplitude of the stimulation (Fröhlich, 2015). This means that, the higher the intensity of stimulation, the larger the mismatch can be between the applied and the endogenous frequency, while still efficiently entraining the ongoing activity. Although we did not match our stimulation frequency to individual peak frequencies in the theta range, our results show that our applied current amplitude of 0.9 mA peak-to-peak was sufficiently large to induce online modulation in the cortical regions being stimulation.

The present results are the first demonstration of the suitability of concurrent tACS-TMS-EEG to assess the relationship between applied current and the cortico-cortical transmission in humans. Three recent studies introduced the combined use of tACS, TMS and electromyography (EMG) recordings as a means to study how tACS applied over the primary motor cortex can modulate MEPs induced by TMS (Guerra et al., 2016; Nakazono et al., 2016; Raco et al., 2016), and provided a demonstration of tACS-phase-dependent changes in local cortical excitability. It should be noted that these approaches to measure responsiveness to tACS through MEPs are limited to the primary motor cortex. The tACS-TMS-EEG method provides an opportunity to comprehensively assess regionally specific responsiveness to tACS, whereas MEPs only can assess the responsiveness to tACS in the primary motor cortex. In addition, these measures have a very limited use for studying network effects. In contrast, the combined use of tACS-TMS-EEG allows for assessing local excitability, as well as network effects at any cortical region. In terms of assessing brain state dependent effects of tACS, these studies are finally limited to motor states, while the tACS-TMS-EEG method would allow to assess the full range of brain state dependent effects of tACS on local excitability and cortico-cortical signaling efficacy.

We show significant differences in TEPs when TMS was applied at the 180° and 360° phase of frontal tACS. These results violate the assumption of a linear relationship between induced current and resulting level of excitability, where excitability would be comparable between the 180° and 360° phases of frontal tACS. Animal models do not support that passage of current through the scalp would introduce appreciable phase-shifts (Logothetis et al., 2007; Opitz et al., 2016). A more probable explanation would be that a shift between applied current and excitability would arise at the neuronal level. Considering the biophysical properties of neurons, neuronal cell membranes are capacitors with a certain time constant, which allows for the temporal summation of postsynaptic potentials. Hence, we could speculate that the resulting change in excitability in the stimulated area, as estimated by TEPs, is explained by accumulated tACS-induced current in neuronal cells during a certain period.

The recent assessments of the relationship between transcranially applied current and excitability in the primary motor cortex in humans have yielded some interestingly divergent findings. In the recently published tACS-TMS-EMG studies, 20 Hz (beta-band) tACS was applied in each study over the primary motor cortex, during which single pulse TMS was applied at 4 phases of the ongoing tACS application. All three studies used the same montage of tACS electrodes, and applied the same intensity of tACS current. However, while one study (Nakazono et al., 2016) reported a linear relationship between the applied current and excitability, the two other studies (Guerra et al., 2016; Raco et al., 2016) reported a shift between applied current and excitability of 90° and 180°, respectively. Our data does not support a linear relationship between applied current and excitability, and in addition cannot be explained by a linear shift neither. These inconsistencies would need to be further systematically assessed. The results however generally advise caution in the interpretation and design of tACS experiments, in particular when aiming to introduce oscillatory activity mimicking endogenous oscillatory activity. Furthermore, a linear modulation of excitability has been the assumption behind using sinusoidal modulation of behavioral performance as evidence for tACS-induced entrainment (for instance Riecke et al., 2015; Stonkus et al., 2016). Both concurrent tACS-TMS-EEG and tACS-TMS-EMG could help to correlate simultaneous behavioral performance with an online measure of excitability modulation, ideally at an individual basis.

Our findings also lend support to the proposed role of inter-regional oscillatory phase-synchrony in modulating inter-regional communication. In the proposed communication-through-coherence (CTC) model (Fries, 2005, 2015), signals are periodically transmitted from a certain region and are bestowed with higher gain in a receiving region depending on the regional phase of excitability. The CTC model then proposes that the dynamically established relation of oscillatory phase between regions can thereby gate signals propagating between these regions, flexibly rendering the inter-regional communication more or less efficient. However, the whole framework of the CTC model strongly relies on the assumption of phase-dependent modulation of signaling efficacy by regional oscillatory activity. This assumption was supported by the correlation between the phase of endogenous local field potential and the firing rate of neurons (Fries et al., 2001). The current study provides a causal link between transmission efficacy and the phase of neural oscillations that supports the framework of the CTC model (Fries, 2005).

In conclusion, our new concurrent tACS-TMS-EEG method allows to address neurophysiological mechanism of tACS-induced neural oscillations. Our results demonstrate that tACS-induced theta oscillations modulate cortical excitability in a phase-dependent manner. This result supports the causal influence of regional oscillatory dynamics on neuronal signaling efficacy.

## Author Contributions

YM conceptualized the experiments. KDF, MN and YM collected data. KDF and YM analyzed data, discussed and interpreted the results and wrote the manuscript.

## Conflict of Interest Statement

The authors declare that the research was conducted in the absence of any commercial or financial relationships that could be construed as a potential conflict of interest.

## Footnotes

^1^http://sccn.ucsd.edu/eeglab/

^2^https://www.r-project.org/
