# Supplementary material for: Phase-Dependent Modulation of Signal Transmission in Cortical Networks through tACS-Induced Neural Oscillations
Source: Front Hum Neurosci. 2017 Sep 27;11:471. doi: 10.3389/fnhum.2017.00471 (PMC5624081; doi:10.3389/fnhum.2017.00471)
Supplement: Supplementary file 1 [file Data_Sheet_1.pdf]

Supplementary Material

Phase-dependent modulation of signal transmission in cortical networks through tACS-induced neural oscillations

Kristoffer D. Fehér [1], Masahito Nakataki [1*], Yosuke Morishima [1,2]

*** Correspondence:**

Yosuke Morishima MD, PhD.

yosuke.morishima@puk.unibe.ch

This file includes

Supplementary Figure S1

Modelling of electric field distribution for the two tACS montages

Supplementary Figure S2

Power spectrum analysis of tACS artifact removal processes

Supplementary Figure S3

TMS evoked potentials during frontal tACS

Supplementary Figure S4

Power spectrum analysis of resting-state EEG data

Supplementary Table 1.

Percentage of remaining trials per condition after bad trial rejection.

Supplementary Table 2.

P-values across time based on one-way repeated measures MANOVA

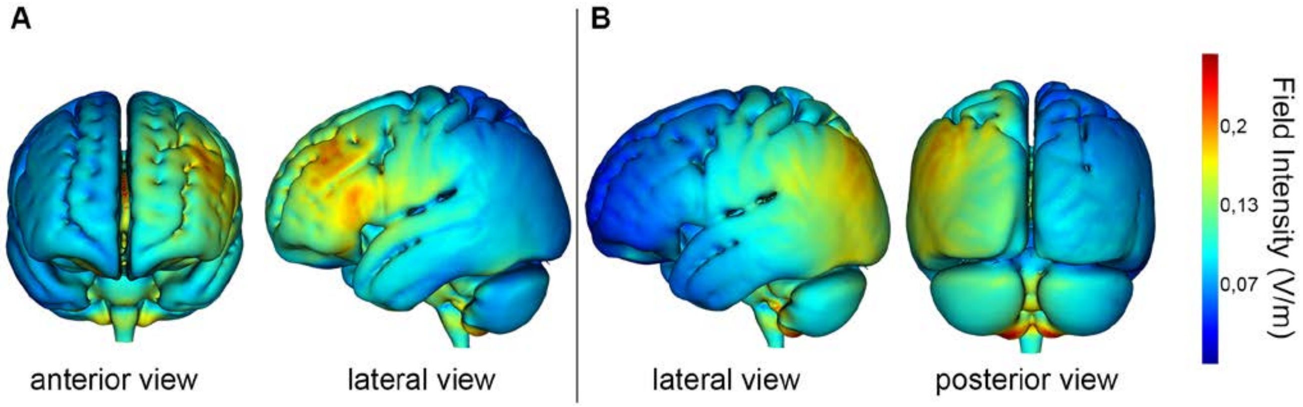

Supplementary Figure S1. Modelling of electric field distribution for the two tACS montages used in the study and a stimulation intensity of 0.9 mA peak-to-peak. (A) Modeled electric field distribution given tACS electrodes centred on F3 and on the neck (approximating our shoulder placement of the return electrode). (B) Modeled electric field distribution given tACS electrodes centred on P3 and on the neck (approximating our shoulder placement of the return electrode). Electric field distribution was modelled by means of a bioelectromagnetic simulator of the current flow into the brain (Soterix HD-Explore, Soterix Medical, NY, USA). The software uses a finite element method to compute the distribution of the electrical field into an adult head model.

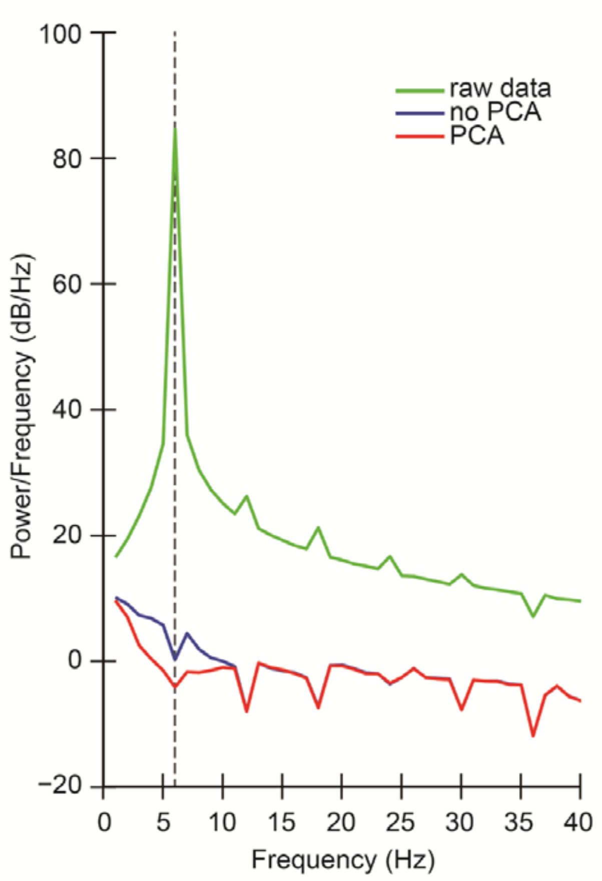

Supplementary Figure S2: Power spectrum of tACS artifact removal processes, obtained from channel Oz of one subject (same as in Fig. 2) during frontal tACS. Power spectrum shows how the tACS induced artifactual frequency components are removed at each step of the pre-processing. Dashed line marks 6 Hz. Power spectrum calculated for unprocessed data (green trace), for data after applying moving average subtraction (blue trace), for data after residual artifact removal through PCA (red trace).

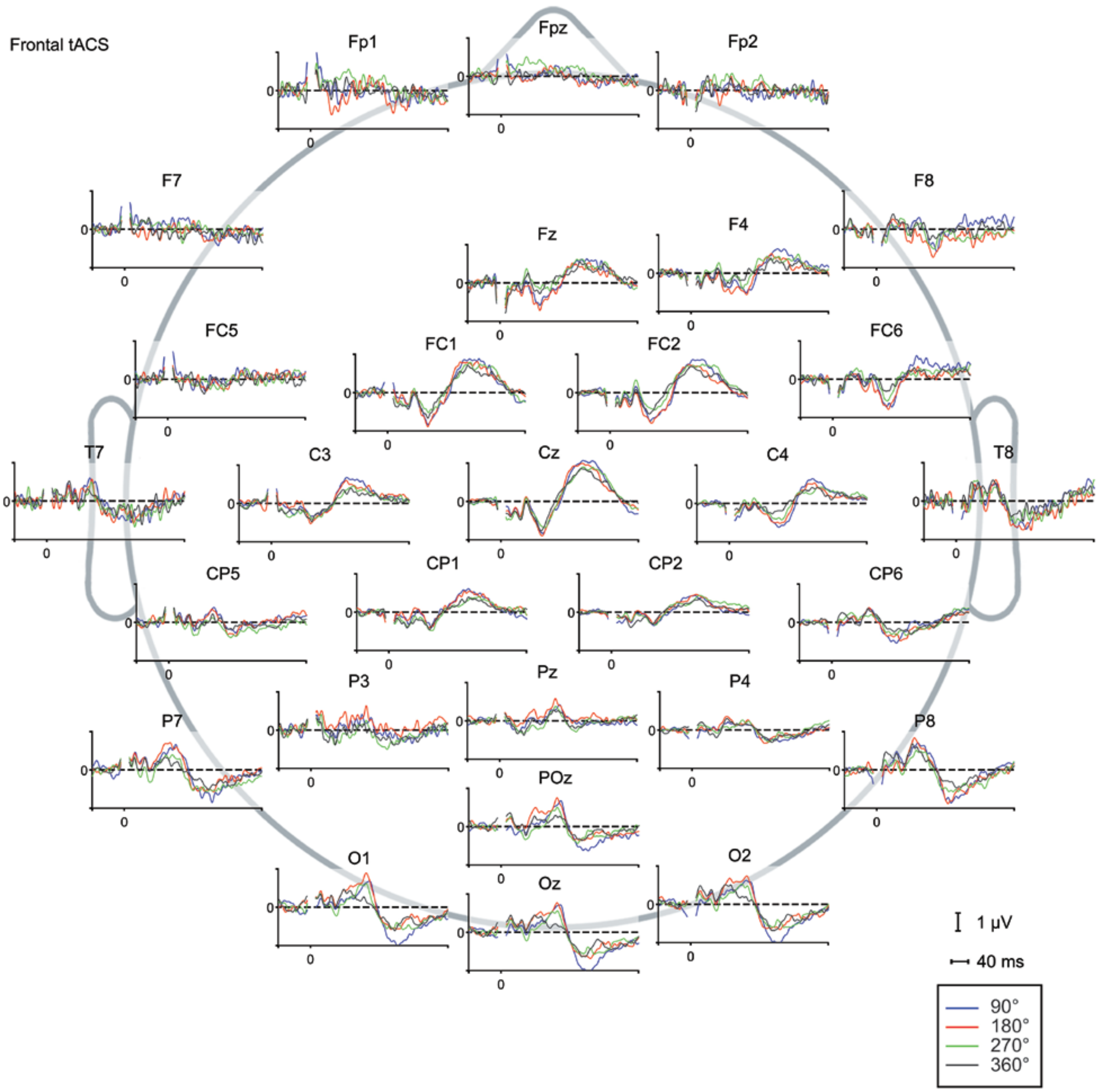

Supplementary Figure S3: Grand-average TEPs across participants, per TMS-delivery phase during frontal tACS.

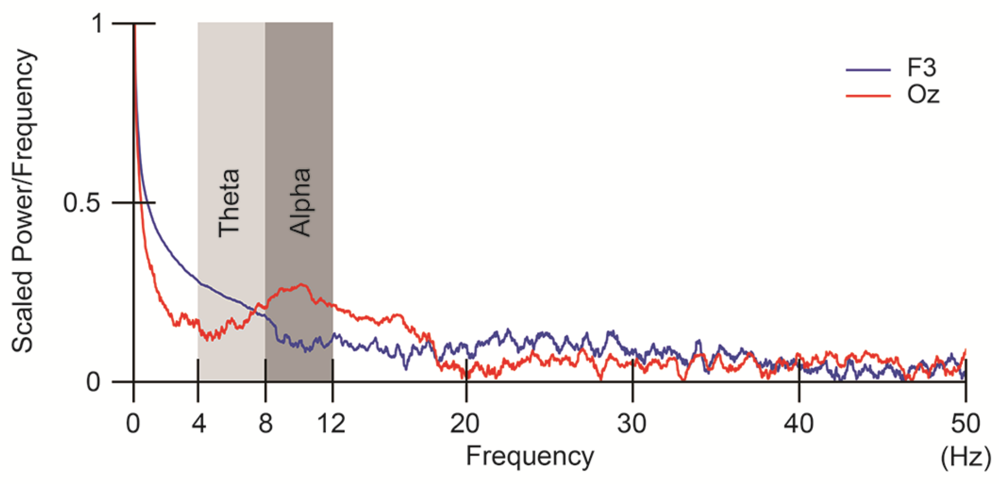

Supplementary Figure S4: Power spectrum across subjects of 1-min resting state EEG, recorded before the stimulation sessions. Eye-blinking was removed from the data using ICA, the data was converted to current source density, and power-spectrum was calculated for each channel. The power-spectrum from the channels F3 and Oz were scaled for comparison (.02 Hz to 50 Hz), and smoothing was applied to the data using a moving average filter, with a span of 25 data-points (corresponding to .5 Hz).

Frontal tACS

TMS condition	90°	180°	270°	360°
	90.94% ±5.72%	90.88% ± 5.51%	91.88% ±5.92%	93.75% ±5.65%

Parietal tACS

TMS condition	90°	180°	270°	360°
	89% ±5.82%	91.19% ±6.84%	91.94% ±6.67%	91.88% ±4.86%

Mean across conditions:
91.43%

Supplementary Table 1. Percentage of remaining trials per condition after bad trial rejection.

FRONTAL RESULTS

MANOVA,
4 phases

Time-window (ms)	20-40	40-60	60-80	80-100	100-120	120-140	140-160	160-180	180-200	200-220	220-240	240-260	260-280	280-300	300-320
Channel:Phase	0.866	0.001	< 0.001	0.005	0.034	< 0.001	0.271	0.001	< 0.001	< 0.001	0.141	0.992	0.909	0.915	0.599
Phase	0.414	0.488	0.165	0.99	0.274	0.733	0.738	0.143	0.064	0.163	0.042	0.474	0.004	0.051	0.06

MANOVA,
180 vs 360

Time-window (ms)	20-40	40-60	60-80	80-100	100-120	120-140	140-160	160-180	180-200	200-220	220-240	240-260	260-280	280-300	300-320
Channel:Phase	0.641	0.054	0.004	0.003	< 0.001	< 0.001	0.005	0.011	0.36	0.271	0.351	0.992	0.775	0.952	0.253
Phase	0.985	0.825	0.125	0.978	0.033	0.463	0.669	0.038	0.873	0.96	0.115	0.986	< 0.001	0.619	0.004

PARIETAL RESULTS

MANOVA,
4 phases

Time-window (ms)	20-40	40-60	60-80	80-100	100-120	120-140	140-160	160-180	180-200	200-220	220-240	240-260	260-280	280-300	300-320
Channel:Phase	0.999	0.907	0.991	0.352	0.002	0.458	0.344	< 0.001	0.007	0.031	0.316	0.949	0.757	0.773	0.416
Phase	0.703	0.538	0.985	0.163	0.89	0.696	0.392	0.391	0.375	0.511	0.418	0.81	0.256	0.679	0.189

MANOVA,
180 vs 360

Time-window (ms)	20-40	40-60	60-80	80-100	100-120	120-140	140-160	160-180	180-200	200-220	220-240	240-260	260-280	280-300	300-320
Channel:Phase	0.948	0.104	0.362	< 0.001	< 0.001	0.054	0.447	< 0.001	0.013	0.002	0.003	0.137	0.038	0.091	< 0.001
Phase	0.307	0.879	0.755	0.443	0.747	0.419	0.101	0.132	0.476	0.591	0.51	0.395	0.512	0.682	0.366

Supplementary Table 2. *P*-values across time based on one-way repeated measures MANOVA, showing interaction effect of the channel and of the delivery-phase of the TMS, and main effect of delivery-phase. Significant results ($p < 0.05$) are marked in colour. The mastoids and channel F3 were excluded from any statistical analysis.
